# Cross-Serotype Protection of a PstS-YidR Fusion mRNA Vaccine Against Systemic Infection and Endogenous Endophthalmitis Caused by Hypervirulent *Klebsiella pneumoniae*

**DOI:** 10.3390/pathogens15090938

**Published:** 2026-09-04

**Authors:** Jiaying Lei, Xinxin Lu, Tiyun Han, Zibing Jin, Qingfeng Liang

**Affiliations:** 1 Beijing Institute of Ophthalmology, Beijing Tongren Eye Center, Beijing Tongren Hospital, Capital Medical University, Beijing 100005, China; leijy2019@163.com (J.L.);; 2 Nanjing Chengshi (TheraRNA) Biomedical Technology Co., Ltd., Nanjing 210000, China

**Keywords:** *Klebsiella pneumoniae*, mRNA vaccine, PstS-YidR, bacteremia, endogenous endophthalmitis, cross-serotype protection

## Abstract

**Background**: Hypervirulent *Klebsiella pneumoniae* (*K. pneumoniae*) easily causes bacteremia and liver abscess, and invades the eye via blood circulation to trigger blinding endogenous endophthalmitis. Widespread multidrug resistance limits antibiotic treatment, while traditional capsular polysaccharide vaccines cannot provide cross-serotype protection. **Methods**: We established a mouse model of intraperitoneal infection-induced endogenous endophthalmitis. BALB/c mice received two intramuscular injections of LNP-encapsulated PstS-YidR fusion mRNA vaccine, followed by challenge with hypervirulent K1 or K2 strains. We monitored body weight, quantified multi-tissue bacterial loads, detected IL-1β, IL-6 and TNF-α, and performed slit-lamp observation and liver/ocular histopathology. **Results**: The vaccine relieved systemic symptoms and weight loss, suppressed bacterial dissemination across peritoneal, blood, liver, lung and eye tissues, and reduced excessive inflammatory factor release. It alleviated intraocular suppurative lesions, preserved ocular structure, and mitigated liver abscess and hepatocellular necrosis, with equal protective efficacy against K1 and K2 and favorable in vivo safety. **Conclusions**: The PstS-YidR fusion mRNA vaccine blocks systemic spread and intraocular invasion of hypervirulent *K. pneumoniae* and alleviates multi-organ inflammatory damage. It serves as a safe candidate vaccine for preventing K1/K2-type hypervirulent Klebsiella infections and endogenous endophthalmitis.

## 1. Introduction

Hypervirulent *Klebsiella pneumoniae* (*K. pneumoniae*) is a Gram-negative pathogen with a rising clinical detection rate and pathogenic risks in recent years. It possesses strong virulence and invasive capacity. Most infections originate from abdominal lesions and pyogenic liver abscesses, which progress to severe systemic disease via hematogenous dissemination. In advanced cases, bacteria cross the blood–ocular barrier and trigger endogenous endophthalmitis [1,2,3]. Clinical data identify this bacterium as the top pathogen of endogenous endophthalmitis across Asia. The disease develops insidiously and progresses rapidly, leading to vitreous suppuration, retinal damage, and irreversible blindness within a short time [4,5,6]. Widespread multidrug resistance further narrows the pool of effective antibiotics. Monotherapy relying on antimicrobials fails to control severe infections and metastatic complications [7,8]. Accordingly, developing novel safe and efficient preventive strategies to block bacterial dissemination and ocular invasion is an urgent demand for infection control.

mRNA vaccines show great promise for preventing bacterial infectious diseases, with advantages including short R&D cycles, stable antigen delivery, non-integrative features, and convenient large-scale production [9,10]. Conventional vaccines against *K. pneumoniae* mainly target capsular polysaccharides. Their protection is strictly serotype-restricted and lacks broad-spectrum activity, making them ineffective against prevalent hypervirulent serotypes in the clinic [11,12,13]. Our team previously constructed a PstS-YidR fusion mRNA vaccine targeting two highly conserved functional proteins shared across Enterobacteriaceae. Prior work has verified its broad-spectrum in vivo protection against multidrug-resistant strains [14]. Although preliminary data confirmed its cross-species efficacy, systematic in vivo evidence is still lacking to clarify its cross-protection against different hypervirulent serotypes of *K. pneumoniae*, especially the clinically dominant K1 and K2 strains.

Most global studies on *Klebsiella* vaccines adopt pulmonary infection models. Animal models recapitulating the full clinical pathological chain (abdominal infection–bacteremia–intraocular invasion) for systemic infection and endogenous endophthalmitis remain scarce [15,16]. No study has systematically validated whether vaccines can block systemic bacterial spread and subsequent intraocular invasion. To fill this research gap, we established a mouse model of systemic infection and endogenous endophthalmitis induced by intraperitoneal injection of hypervirulent *K. pneumoniae*. We systematically evaluated the protective efficacy of the PstS-YidR fusion mRNA vaccine against systemic symptoms, bacterial dissemination, multi-organ injury and intraocular inflammation caused by hypervirulent strains. This study provides experimental evidence to support the clinical translation of this vaccine and the development of prevention strategies for high-risk populations.

## 2. Materials and Methods

### 2.1. Bacterial Strains and Experimental Animals

Two clinical hypervirulent *Klebsiella pneumoniae* strains, serotype K1 (Kp. 8297) and K2 (Kp. 9720), were used in this study. Both isolates were collected from vitreous samples of patients diagnosed with liver abscess complicated by endogenous endophthalmitis. String testing verified their hypermucoviscous phenotype, and PCR confirmed carriage of the virulence gene *rmpA*. Antimicrobial susceptibility assays demonstrated broad-spectrum resistance to cephalosporins, carbapenems, fluoroquinolones and aminoglycosides. Strains were cultured on Columbia blood agar at 37 °C for 16–18 h; single colonies were amplified, and bacterial suspensions were standardized via turbidimetry before in vivo challenge.

SPF male BALB/c mice aged 6–10 weeks (18–25 g) were purchased from Beijing Vital River Laboratory Animal Technology Co., Ltd. (Beijing, China). Mice were housed in IVC systems under controlled conditions (22 ± 2 °C, 50–60% humidity, 12 h light-dark cycle) with ad libitum food and water, and acclimatized for 7 days prior to experiments. All animal operations were approved by the Institutional Review Board of Beijing Tongren Hospital (Approval No: TRLAWEC2023-S007, approval Date: 17 February 2023) and complied with the Guide for the Care and Use of Laboratory Animals, the ARVO Statement for Ophthalmic Animal Research, and national laboratory animal welfare regulations.

### 2.2. Experimental Vaccine

**Figure 1:**
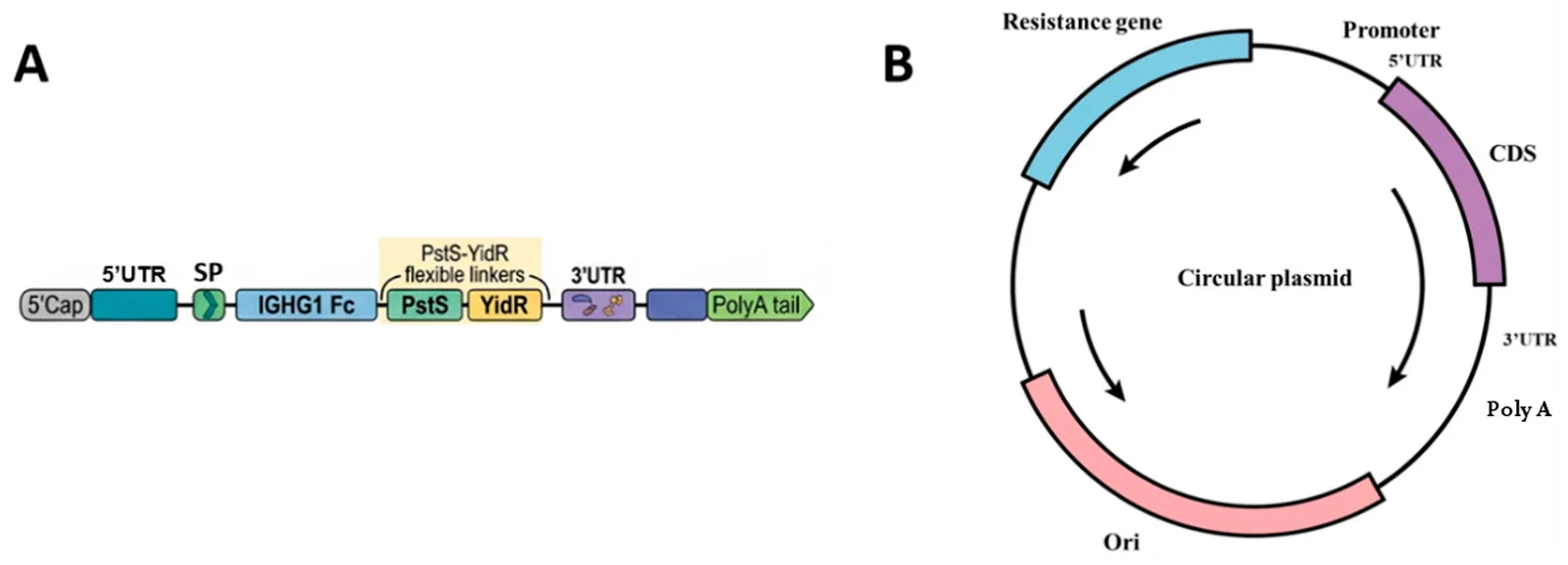
Design and characterization of the PstS-YidR fusion mRNA vaccine. (**A**) Schematic of the mRNA construct containing the T7 promoter, 5′ UTR, human IgG1 Fc (IGHG1), PstS-YidR fusion antigen, STABILON, 6×His tag, 3′ UTR, and poly(A) tail. (**B**) Map of the recombinant plasmid vector carrying regulatory elements for mRNA transcription and expression.

The PstS-YidR fusion mRNA-LNP vaccine used in this study was designed according to the conserved antigen strategy reported in [14]. The dual-antigen mRNA targets two highly conserved proteins of *Klebsiella pneumoniae*, PstS and YidR, with full sequence optimization and stabilization modifications before in vitro transcription and lipid nanoparticle encapsulation. The schematic structure of the mRNA and recombinant plasmid vector is shown in Figure 1.

For plasmid construction, codon-optimized antigen sequences were synthesized by GenScript and inserted into a pUC57 IVT backbone vector carrying a T7 promoter, 5′/3′ UTR and a 110 nt poly(A) tail to improve mRNA stability and translation efficiency. Antigens were fused to the N-terminus of human IGHG1 via flexible GGS linkers, while the C-terminus contained GS linkers, a STABILON-stabilizing motif, and a 6×His tag for protein stabilization and immunodetection. Recombinant plasmids were validated by restriction digestion and Sanger sequencing to confirm intact target inserts and functional regulatory elements.

Linearized plasmid templates were applied for in vitro transcription using the MEGAscript^®^ T7 Transcription Kit (Thermo Fisher Scientific, Waltham, MA, USA) with modified protocols. Co-transcriptional Cap1 capping was performed, followed by enzymatic removal of residual DNA templates and lithium chloride precipitation for mRNA purification. mRNA concentration, purity, and integrity were, respectively, determined by NanoDrop spectrophotometry (NanoDrop 2000; Thermo Fisher Scientific, Waltham, MA, USA) and capillary electrophoresis, with purified mRNA stored short-term at 4 °C.

SM-102-based LNPs were prepared via microfluidic mixing. The lipid mixture (SM-102/DSPC/cholesterol/PEG2000-DMG, molar ratio 50:10:38.5:1.5) was dissolved in ethanol and mixed with mRNA aqueous buffer (20 mM sodium acetate, pH 5.5) at a flow ratio of 95:5, with a lipid:mRNA weight ratio of ~25:1 to drive self-assembly. The finished mRNA-LNP suspension was neutralized to pH 7.0–7.4 using 1 M Tris-HCl, filtered through a 0.22 μm membrane and stored at 2–8 °C. Dynamic light scattering was used to measure particle size, polydispersity index and zeta potential, and the Quant-iT RiboGreen RNA Assay Kit (Thermo Fisher Scientific, Waltham, MA, USA) was adopted to quantify encapsulation efficiency.

### 2.3. Establishment of Mouse Model of Endogenous Endophthalmitis and Screening of Optimal Challenge Dose

Gradient challenge pre-experiments were conducted to screen a stable infectious concentration meeting standardized model criteria. SPF male BALB/c mice aged 6–10 weeks and weighing 18–25 g were used in the pre-experiment.

Extensive screening was performed on clinical *Klebsiella pneumoniae* isolates harvested from patient liver abscess pus, lung tissue and vitreous fluid. Comparative in vitro invasion assays and preliminary in vivo tests confirmed that K1 and K2 strains isolated from patients complicated with concurrent liver abscess and endogenous endophthalmitis possessed markedly stronger hematogenous and intraocular invasive capacity than isolates from other lesion sites. Based on these comparative results, Kp. 8297 (K1) and Kp. 9720 (K2) were selected as the test strains for gradient screening.

For each serotype, four bacterial suspension gradients were set: 1.5 × 10^2^, 1.5 × 10^4^, 1.5 × 10^6^, 1.5 × 10^8^ CFU/mL. Ten mice were allocated to each single-dose subgroup of each serotype (*n* = 10 per strain per gradient). Mice received an intraperitoneal injection of 0.2 mL of the corresponding bacterial suspension and were continuously observed for 4 days post-challenge. On day 4 after infection, peritoneal lavage fluid, whole blood, liver and eyeballs were aseptically collected from surviving animals for bacterial isolation and culture identification.

Uniform criteria for a qualified model: 100% mouse survival within the 4-day observation period, and positive bacterial culture from peritoneal lavage fluid, blood, liver and eyeballs, which could stably reproduce the complete pathological cascade of abdominal infection, bacteremia and secondary intraocular suppurative lesions. Mortality and tissue bacterial positive rates were compared between K1 and K2 across all gradients.

### 2.4. Validation of Prophylactic Protective Efficacy of the Vaccine

#### 2.4.1. Experimental Grouping

All mice used in formal vaccine protection experiments were SPF male BALB/c mice aged 6–10 weeks, consistent with the animal specifications described in Section 2.3. Five experimental groups were set (*n* = 5 per group), including one blank control group, two vaccine subgroups (Vaccine-Kp.1 and Vaccine-Kp.2), and two saline control subgroups (NaCl-Kp.1 and NaCl-Kp.2). The detailed grouping scheme is listed in Table 1. This study focused on prophylactic immunization rather than post-infection therapeutic intervention, so no antibiotic treatment group was included in the design.

#### 2.4.2. Immunization, Challenge and Unified Tissue Sampling Protocol

Mice received two intramuscular immunizations (5 μg vaccine per mouse) into the right hind thigh on day 0 (prime) and day 21 (booster); blank control mice received no injection, and saline groups received equal-volume sterile saline. On day 35, all challenged mice were intraperitoneally injected with 1.5 × 10^4^ CFU/mL bacterial suspension (the optimal dose screened in Section 2.3). General symptoms and ocular manifestations were recorded daily for 3 days post-challenge. All animals were euthanized via CO_2_ asphyxiation on day 38, followed by standardized tissue dissection with unified sample allocation rules to avoid cross-sample conflict for multiple detections:

(a) Peritoneal lavage fluid and whole blood were collected for bacterial culture, 16S rRNA sequencing and CBA cytokine testing.
(b) Bilateral eyeballs were separated: the right eyeball was homogenized for bacterial load and cytokine detection, while the left eyeball was fixed in special FAS fixative for paraffin embedding and H&E staining.
(c) Whole livers and left lung tissues were photographed for gross morphology and split equally into two parts. One aliquot was homogenized for bacterial and cytokine assays, and the other liver fragment was fixed in 4% paraformaldehyde for pathological staining; lung tissue was discarded without histological processing. Because the lung was already quantitatively evaluated by 16S rRNA sequencing and cytokine profiling, dedicated lung histology was not performed. Histopathological effort was instead focused on the eyeball and liver—the two organs central to the endogenous endophthalmitis pathogenic chain.

All tissue samples were processed without cross-contamination for subsequent bacterial quantification, cytokine detection, and histopathological observation by a blinded pathologist.

#### 2.4.3. In Vivo Observation and Multi-Index Detection

Daily monitoring covered mouse mental status, motor activity, hair condition, respiration and ocular secretions, with fixed-time body weight measurement recorded from day 0 to day 38. At the experimental endpoint, slit-lamp microscopy at 40× magnification was used to observe ocular lesions, and a semi-quantitative scoring system (0–3 per indicator, total 0–12 points) was applied to evaluate conjunctival congestion, corneal opacity, iris inflammation and vitreous opacity. Two blinded researchers scored independently, and averaged values were used for statistics.

For qualitative verification of bacterial growth and identification of the dominant colonizer, serially diluted tissue homogenates were spread on Columbia blood agar, while 16S rRNA high-throughput sequencing was performed as the per-animal quantitative readout of relative Klebsiella pneumoniae abundance across organs. The cytometric bead array (CBA) method was used to measure tissue concentrations of IL-1β, IL-6 and TNF-α, with technical duplicates set for all samples.

#### 2.4.4. Statistical Analysis

All statistical analyses were performed using GraphPad Prism 10.0. Measurement data were presented as mean ± standard deviation (SD). Body-weight trajectories (days 0–38) from the same animals are longitudinal repeated measures and were analyzed by repeated-measures ANOVA (RM-ANOVA) with Šidák correction; for single-timepoint multi-group comparisons of bacterial burden—including 16S rRNA relative abundance and culture colony counts (non-normally distributed, many zeros in blank controls)—the Mann–Whitney U test (two-group) or Kruskal–Wallis H test (multi-group) was used on untransformed values, and exact *p* values are reported; the same non-parametric approach (Mann–Whitney U with Šidák correction) was applied to intraocular cytokine concentrations (IL-1β, IL-6, TNF-α); ordinal ocular inflammation scores were analyzed by Kruskal–Wallis H test. Technical duplicates were averaged; *n* = 5 denotes biological replicates per group. Significance: *** *p* < 0.001, ** *p* < 0.01, * *p* < 0.05; ns = not significant.

## 3. Results

### 3.1. Establishment of the Endogenous Endophthalmitis Mouse Model and Determination of the Optimal Challenge Dose

Current animal infection models for hypervirulent *Klebsiella pneumoniae* (hvKP) predominantly focus on pulmonary infection and isolated liver abscess models. Studies recapitulating the complete clinicopathological cascade of primary intraperitoneal infection, bacteremia, hematogenous dissemination, and subsequent endogenous endophthalmitis remain relatively scarce. In the present study, clinically isolated hypervirulent hvKP strains K1 (Kp.8297) and K2 (Kp.9720) were employed. A preliminary experiment was performed with four graded challenge doses, with 10 BALB/c mice allocated to each group, and continuous survival monitoring was conducted for four consecutive days. Survival data of mice in each group are summarized in Table 2.

As shown in Table 2, the survival rates of mice challenged with 1.5 × 10^8^ CFU/mL and 1.5 × 10^6^ CFU/mL were 20% and 60%, respectively. No mortality was observed in the group receiving 1.5 × 10^2^ CFU/mL within the 4-day observation period; however, bacterial culture of ocular tissues yielded negative results in all animals after necropsy on day 4. For the 1.5 × 10^4^ CFU/mL challenge group, both K1 and K2 strain-infected mice exhibited a 100% survival rate over 4 days, with 100% positive bacterial culture rates detected in peritoneal lavage fluid, peripheral blood, liver and eye tissues. No statistically significant differences in mouse mortality or bacterial colonization positivity rates across various tissues were identified between K1 and K2 strain-infected groups at the identical infectious dose. Accordingly, the challenge concentration of 1.5 × 10^4^ CFU/mL was selected for subsequent formal experiments. This non-lethal dose was selected because the primary endpoint—ocular colonization and intraocular inflammation—requires survivors for assessment. Among the four screened doses, 1.5 × 10^2^ CFU/mL was non-lethal but failed to colonize ocular tissues, whereas 1.5 × 10^6^ and 1.5 × 10^8^ CFU/mL caused 40% and 80% mortality, precluding ocular evaluation; only 1.5 × 10^4^ CFU/mL met both predefined criteria (100% survival and 100% multi-tissue, including ocular colonization).

### 3.2. Effects of the Vaccine on General Conditions and Body Weight Changes in Mice

A prior study demonstrated that the PstS-YidR fusion mRNA vaccine (KV3) provided robust protection against pulmonary *K. pneumoniae* infection, effectively lowering lung bacterial burden and alleviating pulmonary inflammatory lesions [14]. Nevertheless, its systemic protective capacity against hypervirulent K1/K2 strains with hematogenous spread has not been characterized. We thus monitored the general conditions and body weight of mice to assess the systemic protective effect of this vaccine.

No deaths occurred in any group over the entire experimental timeline, and no vaccine-associated local or systemic adverse events were recorded during prime-boost immunization, with comparable growth performance across all groups. Post-challenge clinical manifestations differed sharply between treatment arms: vaccine-immunized mice developed only temporary mild lethargy and minor eye discharge that resolved within 3 days, whereas saline-treated animals displayed persistent severe systemic infection symptoms including anorexia, piloerection, tachypnea and abundant purulent ocular secretions.

**Figure 2:**
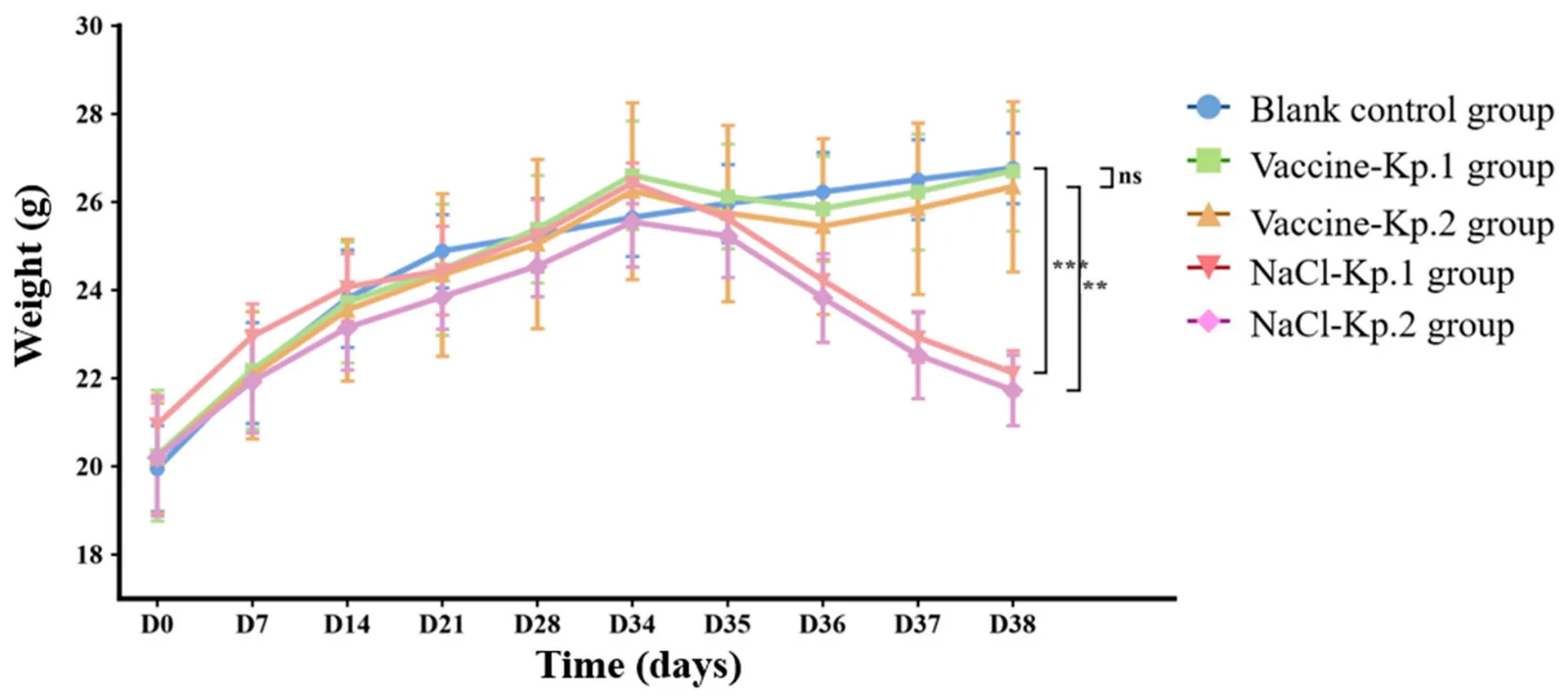
Body weight dynamics of BALB/c mice from day 0 to day 38. Animals received prime-boost intramuscular immunization on day 0 and day 21, then intraperitoneal K1/K2 strain challenge on day 35. Daily weight data of five groups were recorded. Values represent means ± SD, *n* = 5 per group. Statistical analysis: repeated-measures ANOVA with Šidák correction. *** *p* < 0.001, ** *p* < 0.01, ns = not significant.

Body weight dynamics across day 0–38 are presented in Figure 2. All groups gained weight equivalently throughout the immunization phase prior to bacterial challenge. Marked intergroup disparities emerged after intraperitoneal challenge on day 35: blank control mice maintained steady weight growth; vaccine recipients experienced a brief mild weight loss followed by rapid recovery; saline-challenged mice underwent sustained, pronounced weight reduction, with weight loss magnitude markedly higher than the corresponding vaccine groups (** *p* < 0.01). Notably, the extent of weight fluctuation did not differ significantly between K1 and K2 challenge subgroups (*p* > 0.05), demonstrating consistent systemic protection against both major hypervirulent serotypes.

### 3.3. Effects of the Vaccine on Gross Liver Morphology in Mice

**Figure 3:**
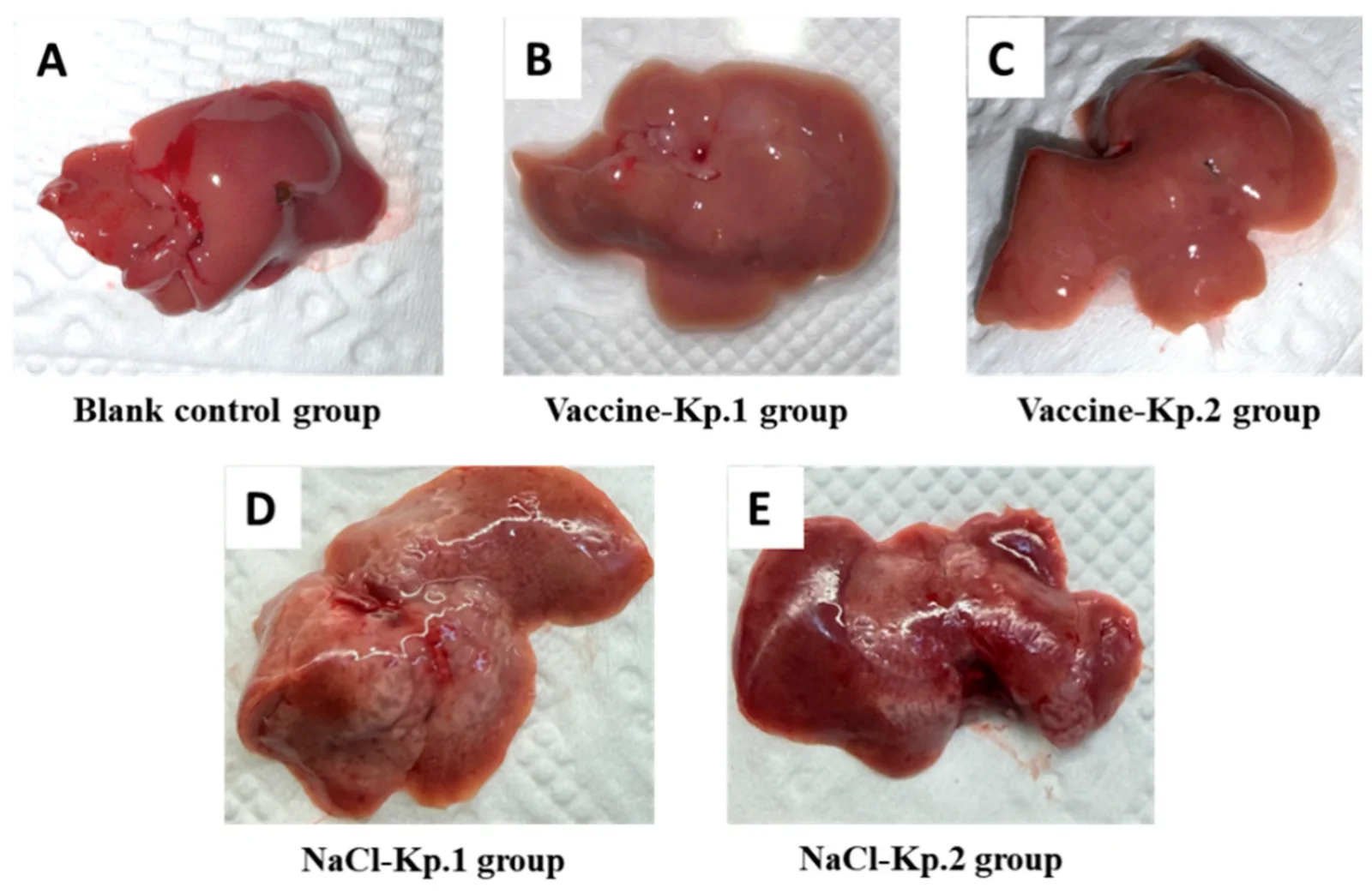
Gross morphological appearance of mouse liver tissues harvested at day 38 post-challenge. (**A**–**E**) Representative images of liver tissues in each group. Compared with the normal liver in group (**A**), mild edema is observed in groups (**B**,**C**), while multiple obvious liver abscesses are present on the surface of livers in groups (**D**,**E**).

Figure 3A–E show the gross morphology of the liver in mice of the five groups after dissection and rinsing, with obvious inter-group differences observed by naked eye: the blank control group (Figure 3A) had regular liver morphology, smooth surface, uniform light red color, and soft and elastic texture, consistent with the normal gross characteristics of mouse liver. The vaccine-immunized challenge groups (Figure 3B,C) showed slightly edematous liver appearance, mild volume enlargement, and slightly pale surface color, with no obvious abscess, necrotic foci, or bleeding spots, indicating no significant pathological damage to the liver. In contrast, the saline control challenge groups (Figure 3D,E) presented marked pathological changes, with multiple grayish-white liver abscesses of varying sizes and clear boundaries on the surface; some abscesses were slightly protruding and hard in texture, indicating severe infectious injury to the liver.

### 3.4. Inhibitory Effects of the Vaccine on Ocular Inflammation in Mice

To evaluate vaccine-mediated ocular protection against hematogenous invasive hypervirulent *K. pneumoniae* (hvKp)-induced destructive endogenous endophthalmitis, slit-lamp microscopy was performed on day 38.

**Figure 4:**
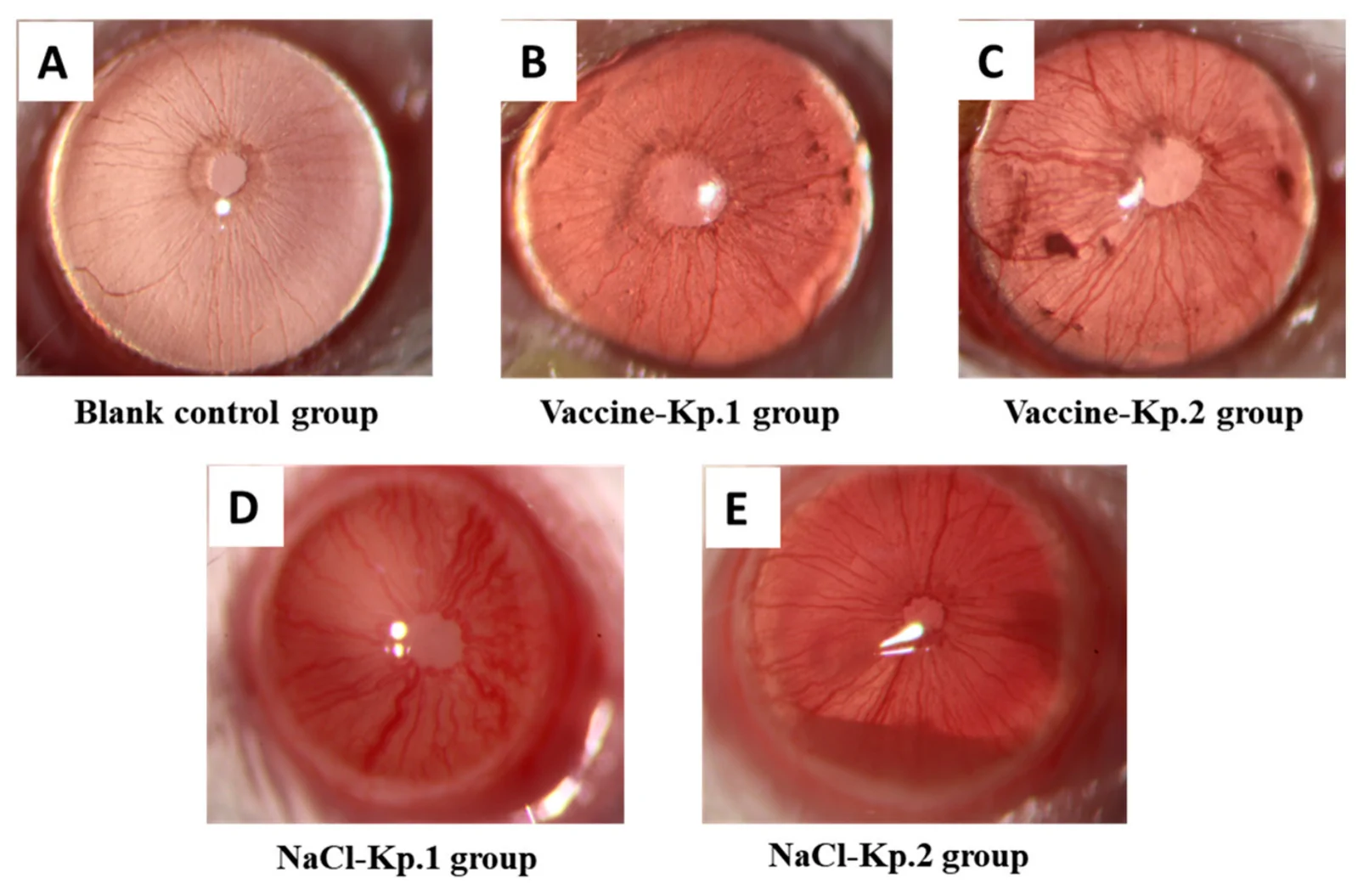
Representative slit-lamp microscopy of mouse eyes (40× magnification). Compared with group (**A**), mild corneal edema and slight vasodilation and congestion of iris vessels are seen in groups (**B**,**C**). Severe corneal edema, marked vasodilation and congestion of iris vessels, and obvious exudation are observed in groups (**D**,**E**). Diffuse grayish-white exudates fill the posterior vitreous cavity in group (**D**), and thick purulent exudates occupy the inferior 1/4 of the vitreous cavity in group E.

Slit-lamp examination (Figure 4) revealed stark differences in ocular pathology. Mice in the blank control group (Figure 4A) displayed normal ocular structures. In contrast, all mice in the saline control challenge groups (Figure 4D,E) exhibited classic signs of severe endogenous endophthalmitis, including marked conjunctival congestion, diffuse corneal opacity, iris hyperemia with blurred texture, and purulent exudation in the vitreous cavity. Vaccine-immunized mice (Figure 4B,C) showed only mild and localized inflammation, with only 30–40% exhibiting slight conjunctival congestion and minor iris vessel dilation, but no corneal opacity or vitreous exudation, effectively preventing pathological changes in purulent endophthalmitis.

**Figure 5:**
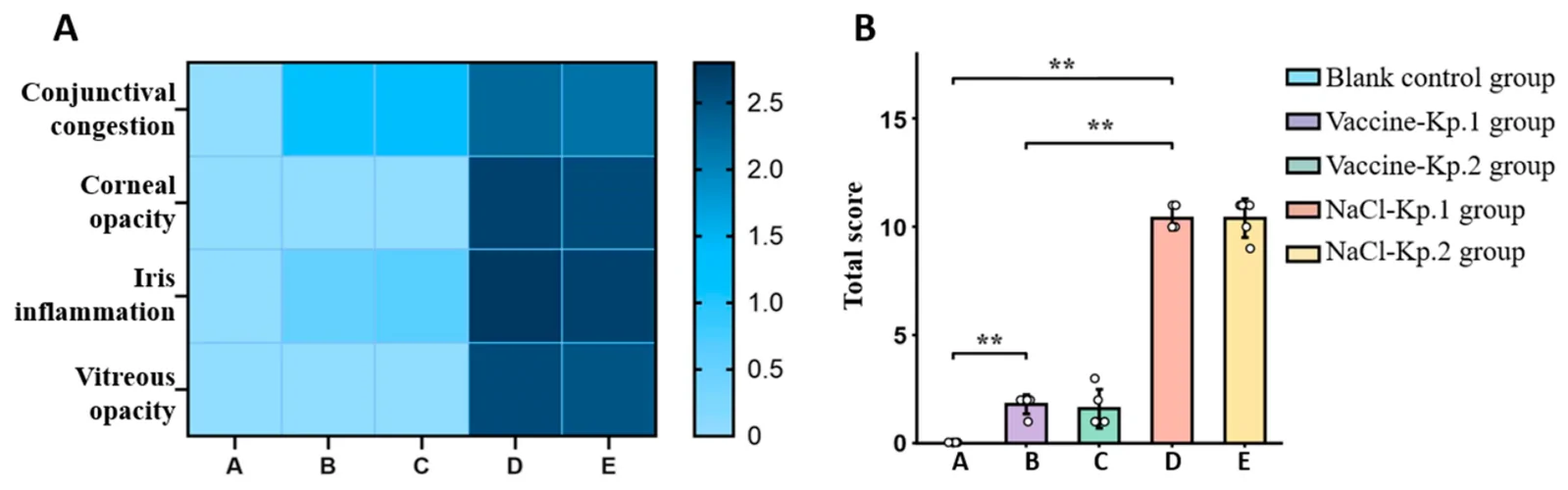
Semi-quantitative scoring for ocular inflammatory lesions. (**A**) Heatmap of individual ocular inflammation scores. (**B**) Total ocular inflammation scores (means ± SD, *n* = 5 per group). Statistics: Kruskal–Wallis test for ordinal scores (**A**); Mann–Whitney U test with Šidák correction for totals (**B**). ** *p* < 0.01.

Semi-quantitative scoring of ocular inflammation (conjunctival congestion, corneal opacity, iris inflammation, and vitreous opacity) was performed (Table 3 and Figure 5). All ocular inflammation scores were assigned by an investigator who was blinded to group allocation, including serotype identity (K1 vs. K2); scoring followed the predefined 0–3 ordinal rubric (Table 3) applied uniformly across all five groups. Heatmap analysis (Figure 5A) visually confirmed that saline control groups (D, E) had widespread high inflammation scores, indicative of severe endophthalmitis, while vaccine groups (B, C) showed scores comparable to the blank control (A). Quantitative analysis of total inflammation scores (Figure 5B) confirmed that saline control groups had extremely high scores post-challenge, with no significant difference between K1 and K2 serotypes (*p* > 0.05), suggesting similar ocular pathogenicity. In contrast, vaccine-immunized groups exhibited drastically reduced inflammation scores, which were highly similar to the blank control group and significantly lower than their respective saline controls (** *p* < 0.01). Demonstrating that the vaccine effectively suppressed ocular tissue injury induced by both K1 and K2 hvKp serotypes.

### 3.5. Inhibitory Effects of the Vaccine on Bacterial Load in Multiple Tissues

**Figure 6:**
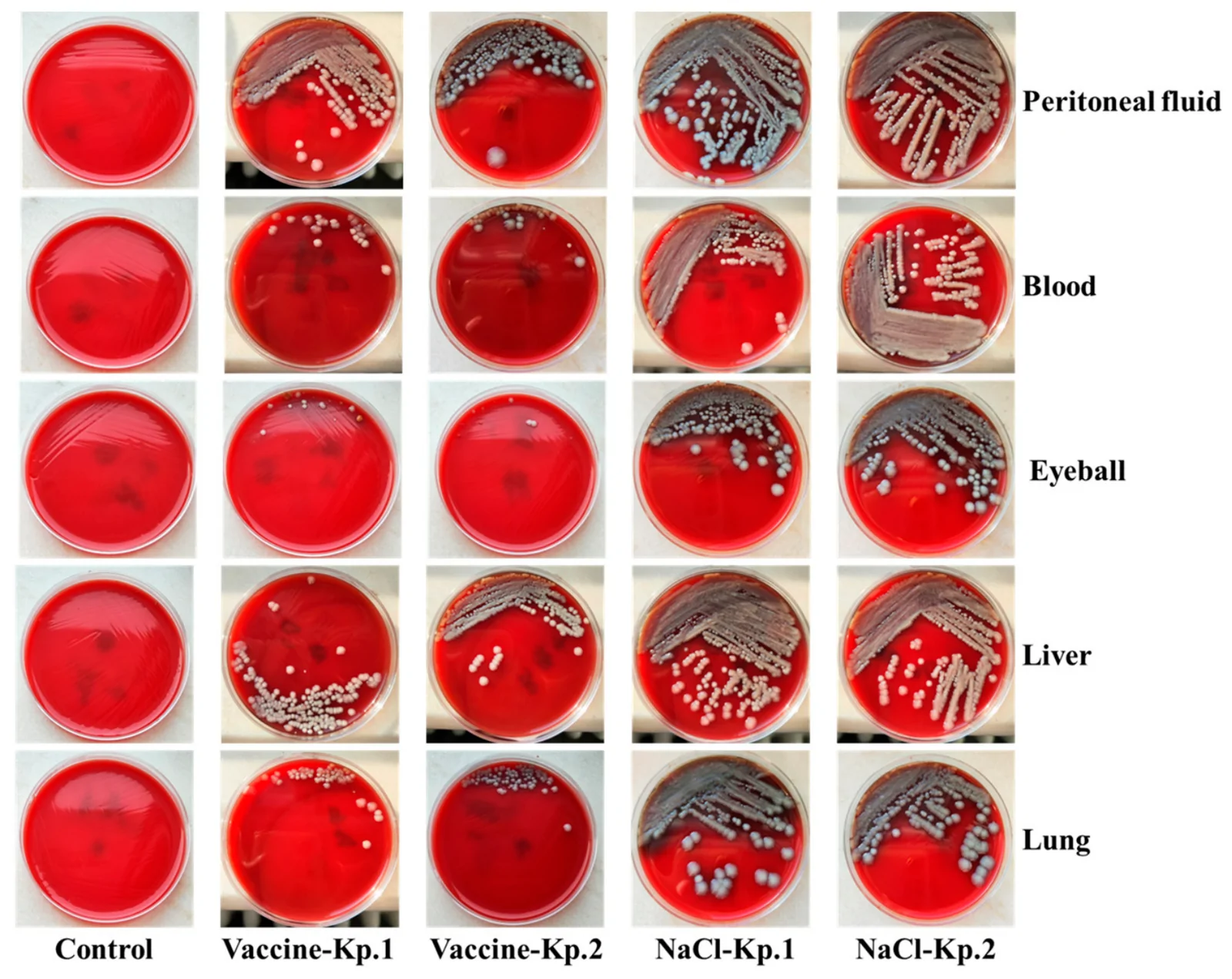
Bacterial culture of multiple mouse tissues. Round, raised, grayish-white mucoid colonies on each plate represent cultured *K. pneumoniae*, and the colony count is proportional to the bacterial load in the corresponding tissue. Representative plates are shown to qualitatively confirm bacterial growth and the dominant colonizer.

**Figure 7:**
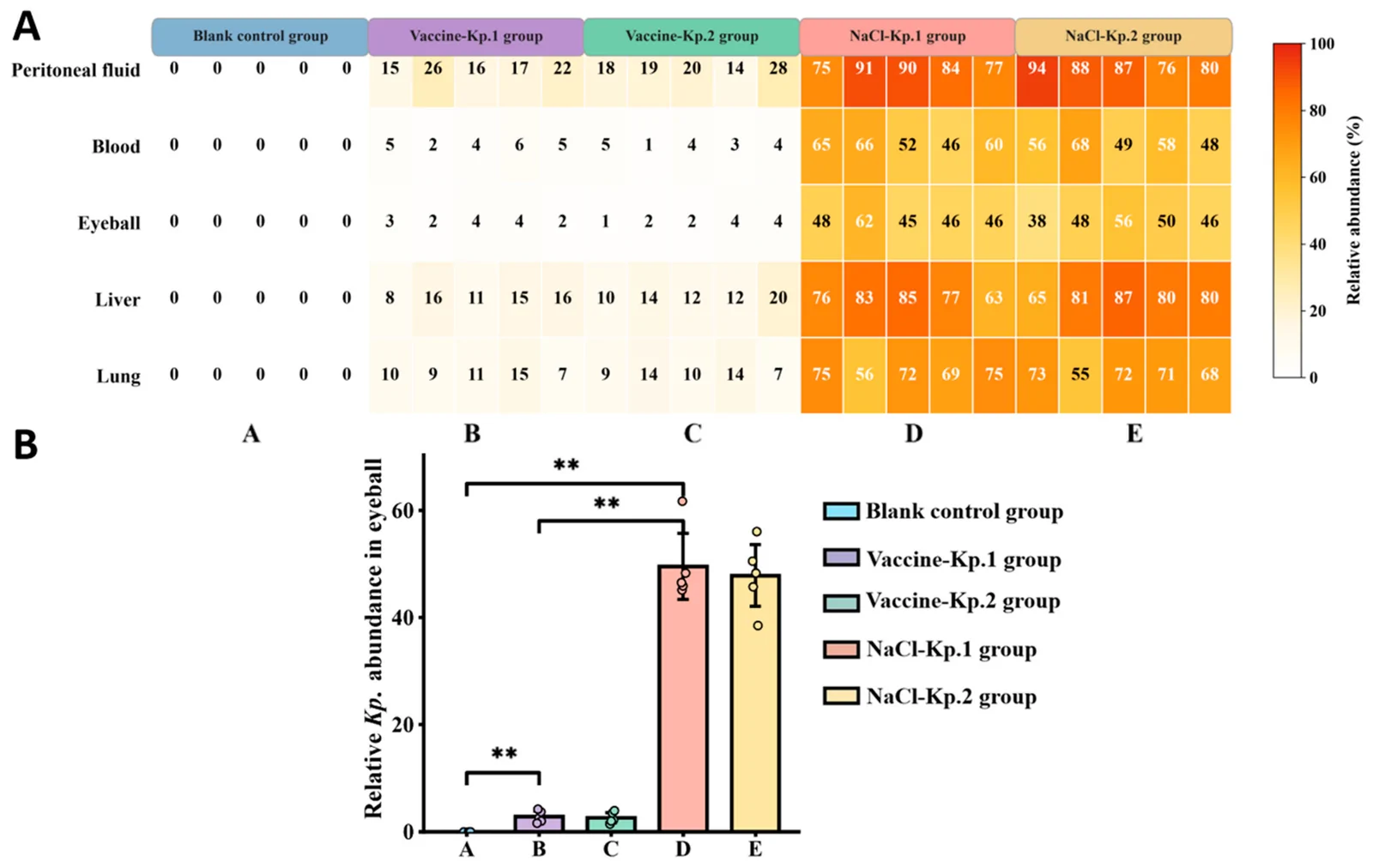
Individual-animal *K. pneumoniae* burden across tissues and the eye, quantified by 16S rRNA sequencing (*n* = 5 per group; relative abundance = percentage of *K. pneumoniae* among total bacterial 16S rRNA reads). (**A**) Heatmap of per-mouse relative abundance across five tissues, with the value shown in each cell. (**B**) Intraocular (eyeball) relative abundance per mouse (dots = individuals, bars = mean ± SD). Exact two-sided Mann–Whitney U test with Šidák correction: blank vs. Vaccine-Kp.1, *p* = 0.0079 (**); Vaccine-Kp.1 vs. NaCl-Kp.1, *p* = 0.0079 (**); blank vs. NaCl-Kp.1, *p* = 0.0079 (**). ** *p* < 0.01.

Peritoneal lavage fluid, blood, liver, lung, and eyeball tissues of mice in each group were aseptically collected for bacterial culture and 16S rRNA sequencing. No K. pneumoniae was recovered from any tissue of the blank control group (0% relative abundance). In the saline-challenged groups, K. pneumoniae was the dominant colonizer in every sampled tissue (mean relative abundance 84.4 ± 6.6% peritoneal fluid, 57.0 ± 7.3% blood, 77.7 ± 7.5% liver, 68.7 ± 6.9% lung, 48.7 ± 5.9% eyeball; 100% culture positivity). Vaccination reduced the relative abundance in all tissues—19.5 ± 4.2% peritoneal fluid, 3.8 ± 1.4% blood, 13.4 ± 3.2% liver, 10.5 ± 2.7% lung, and 2.7 ± 1.0% eyeball—corresponding to 4.3-, 15.0-, 5.8-, 6.5- and 18.0-fold lower burdens than saline controls (all *p* = 0.0079, *p* < 0.01, exact two-sided Mann–Whitney U test; all *p* = 0.0079, *p* < 0.01, exact two-sided Mann–Whitney U test; representative culture plates are shown in Figure 6 as a qualitative corroboration, and individual animal data are presented in Figure 7). These results indicate that the vaccine effectively blocked bacterial invasion from the peritoneal cavity into the blood, inhibited systemic dissemination, and reduced the risk of intraocular invasion.

16S rRNA high-throughput sequencing provided an orthogonal, per-animal quantification of relative K. pneumoniae abundance across tissues. As shown in Figure 7A, the tissue-specific relative-abundance profile was consistent with the culture results, and the intraocular relative abundance in vaccine groups was significantly lower than in saline-challenged groups. As shown in Figure 7B, per-animal relative abundance in eyeball tissues revealed no statistically significant difference between group B (Vaccine + Kp.1) and group C (Vaccine + Kp.2), which is consistent with—and corroborates—the equivalent cross-serotype protection also supported by the integrated clinical, histopathological and cytokine endpoints.

### 3.6. Regulatory Effects of the Vaccine on Inflammatory Factors in Multiple Tissues

**Figure 8:**
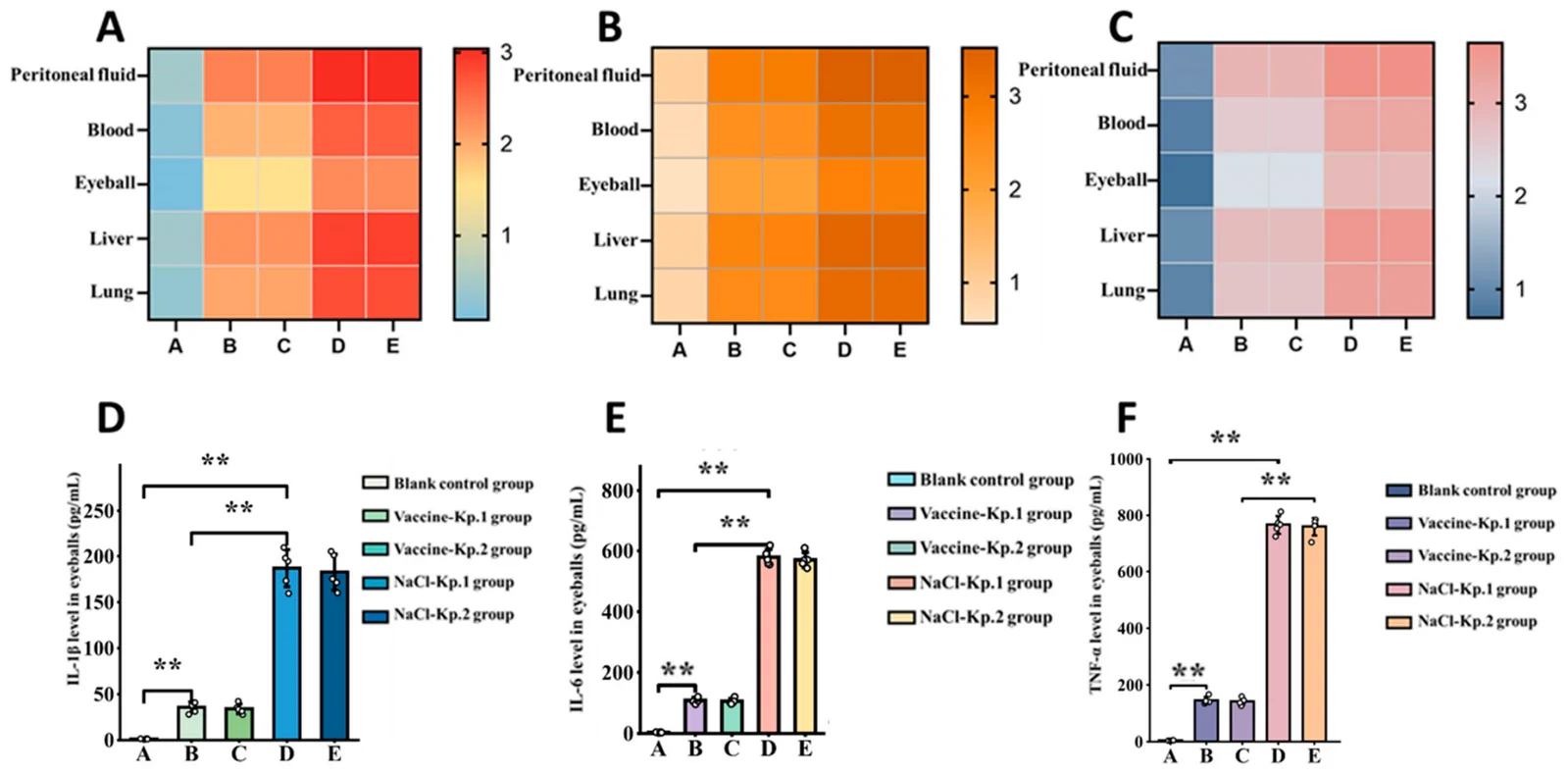
Expression of IL-1β, IL-6, and TNF-α in multiple tissues. (**A**–**C**) Heatmaps of log10-transformed cytokine levels. (**D**–**F**) Bar charts of intraocular cytokine concentrations. Data are means ± SD, *n* = 5 per group. Statistical analysis: Mann–Whitney U test with Šidák correction. ** *p* < 0.01.

Cytometric bead array (CBA) was adopted to quantify tissue IL-1β, IL-6 and TNF-α at day 38 post-challenge across peritoneal lavage fluid, blood, eyeball, liver and lung (Figure 8A–F). Heatmaps (Figure 8A–C) illustrated obvious group-level differences in cytokine abundance: blank control mice sustained basal cytokine levels with uniformly low signal intensity across all tissues, while saline-challenged groups displayed extensive high-intensity signals throughout sampled organs, reflecting robust systemic inflammatory activation post-infection. By contrast, vaccine recipients presented markedly diminished cytokine signals, with faint staining limited only to peritoneal lavage fluid, closely resembling the basal expression pattern of blank controls.

All three cytokines exhibited identical tissue expression gradients: peritoneal lavage fluid > liver > lung > blood > eyeball. Though the eye, as a distant target organ, harbored lower baseline cytokine concentrations, intraocular pro-inflammatory mediators in saline groups remained 4–6-fold higher than those in vaccine groups. Bar graphs of ocular cytokine levels (Figure 8D–F) further verified consistent intergroup variation across IL-1β, IL-6 and TNF-α. The three cytokines in ocular tissues of vaccinated mice merely accounted for 18–19.5% of the levels measured in saline controls (*p* < 0.01, exact two-sided Mann–Whitney U test).

No significant inter-serotype differences were observed in cytokine concentrations across all tested tissues between Vaccine-Kp.1 and Vaccine-Kp.2 groups, nor between NaCl-Kp.1 and NaCl-Kp.2 subgroups (*p* > 0.05). This uniform anti-inflammatory capacity against K1 and K2 hypervirulent strains supports the cross-serotype design of the fusion mRNA vaccine.

### 3.7. Protective Effects of the Vaccine on Histopathological Injury of Eyeball and Liver

**Figure 9:**
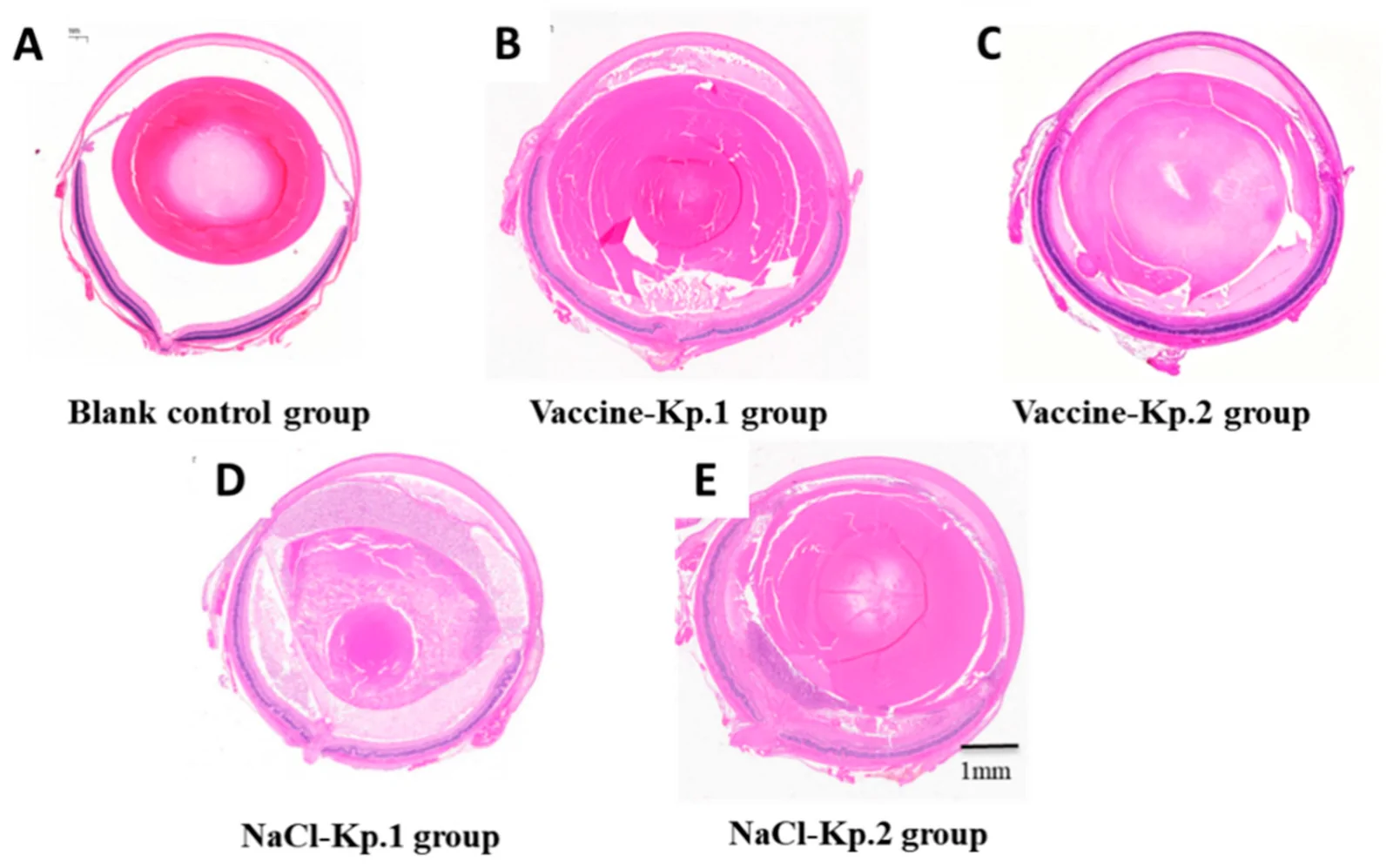
Histopathology of mouse eyes (H&E staining, 400×). Group (**A**) shows normal ocular anatomical structure, clear aqueous humor, no exudation in the vitreous cavity, and clear boundaries between ocular tissues. Vaccine groups (**B**,**C**) present only protein-like exudation and scattered inflammatory cells in the anterior chamber and vitreous cavity, with intact overall ocular morphology and no severe chamber compression. NaCl groups (**D**,**E**) exhibit typical panophthalmitis: the anterior chamber and vitreous cavity are filled with dense inflammatory exudates, tissue boundaries are blurred, and chamber compression is obvious.

**Figure 10:**
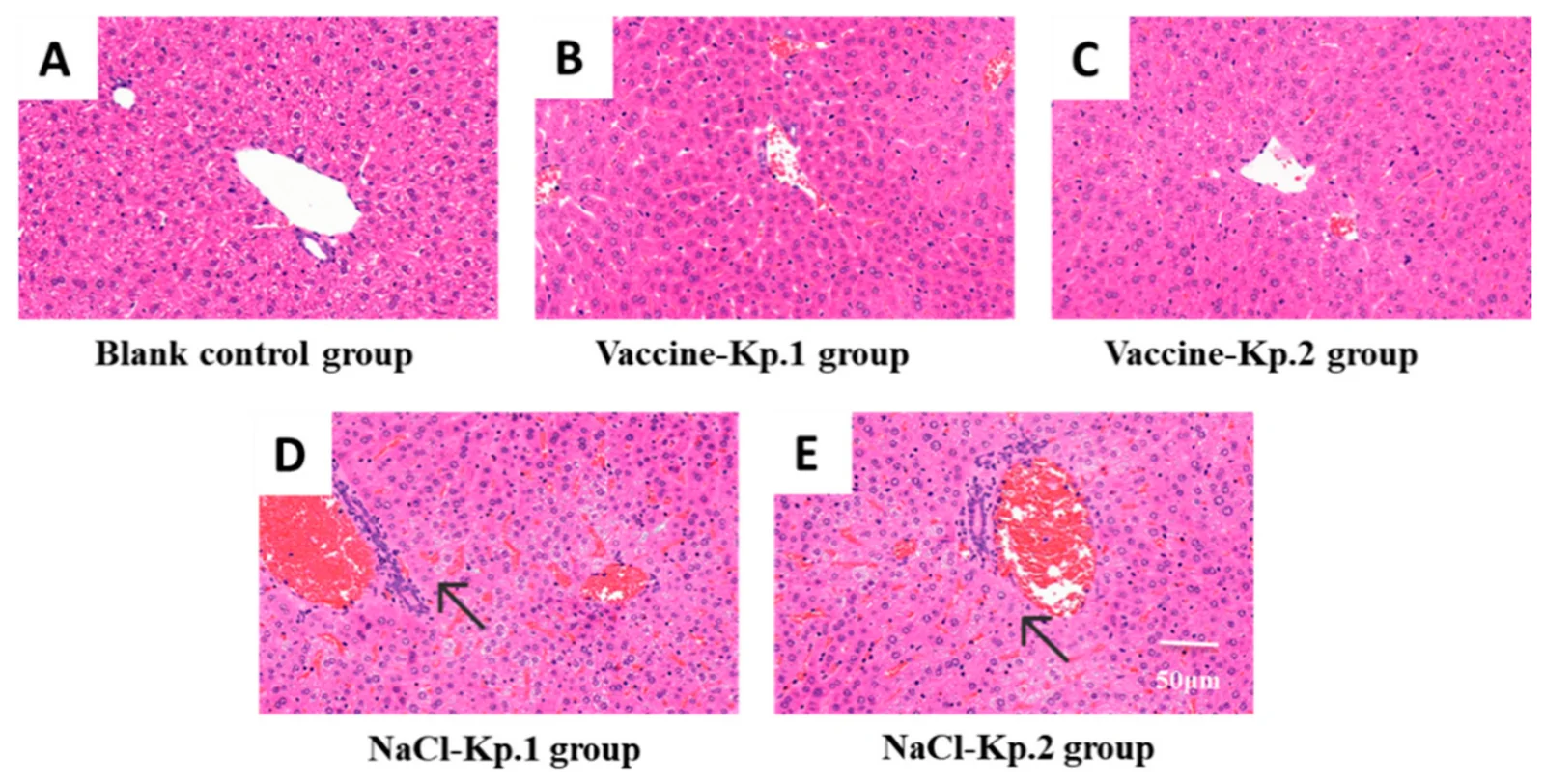
Histopathology of mouse livers (HE staining, 400×). Blank control group (**A**): Intact liver structure, clear hepatic lobule contour, uniform hepatocyte morphology, regular hepatic sinusoids without inflammatory infiltration. Vaccine-Kp.1 (**B**) and Vaccine-Kp.2 (**C**) groups: Only focal mild hepatocyte edema, slightly narrow hepatic sinusoids, occasional scattered inflammatory cells, no abscesses or necrotic foci. NaCl-Kp.1 (**D**) and NaCl-Kp.2 (**E**) groups: Extensive lytic necrosis of hepatocytes, severe dilation and congestion of hepatic sinusoids, massive inflammatory cell infiltration around necrotic foci, and blurred tissue boundaries. Arrows in the NaCl-Kp.1 (**D**) and NaCl-Kp.2 (**E**) groups indicate the necrotic foci with surrounding massive inflammatory cell infiltration.

Hematoxylin–eosin (H&E) staining of paraffin-embedded eyeball and liver sections was conducted to assess tissue structural damage induced by systemic hvKp infection (Figure 9 and Figure 10).

For ocular histopathology (Figure 9): Blank control eyes (Figure 9A) displayed intact global anatomy, continuous corneal epithelium, ordered stroma, and well-defined retinal layers without inflammatory infiltrates. Saline-challenged mice (Figure 9D,E) suffered severe pan-ocular damage, including corneal edema, disorganized stromal fibers, disrupted retinal architecture, massive inflammatory cell infiltration, and vitreous purulent exudates. In contrast, vaccine-immunized animals (Figure 9B,C) retained nearly complete ocular structures, only accompanied by mild corneal edema and sparse inflammatory cell recruitment, with far milder pathological lesions than saline controls.

Liver histopathological analysis (Figure 10) yielded consistent protective trends. Livers from blank mice (Figure 10A) exhibited intact hepatic lobules, uniform hepatocyte morphology, regular hepatic sinusoids, and no inflammatory infiltration. Vaccine groups (Figure 10B,C) only presented focal mild hepatocellular edema and slight sinusoidal compression, lacking extensive parenchymal necrosis. Saline-challenged livers (Figure 10D,E) developed typical acute suppurative lesions, featuring widespread hepatocyte lytic necrosis, massive perifocal neutrophil aggregation, severely dilated congested sinusoids and disrupted sinusoidal walls, which reflected irreversible liver microcirculation impairment and tissue destruction triggered by hypervirulent *K. pneumoniae*.

## 4. Discussion

Hematogenous dissemination serves as the core pathogenic mechanism of endogenous endophthalmitis triggered by hypervirulent *Klebsiella pneumoniae* (hvKp). This disease develops insidiously and progresses rapidly, imposing substantial clinical burdens on infection control and ophthalmic emergency management across the Asia-Pacific region [17,18]. Primary lesions, including intra-abdominal infection and pyogenic liver abscess, are the major origins of hvKp infection; sustained bacteremia enables bacteria to cross the blood–ocular barrier, which eventually causes intraocular suppurative inflammation and irreversible retinal damage [19]. Regrettably, combined antibiotic and surgical intervention cannot effectively block disease progression in severe cases [19]. The rampant prevalence of carbapenem-resistant hvKp strains further restricts the scope of available clinical antibacterial agents [8,20]. Under such circumstances, developing prophylactic vaccines that block bacterial spread and reduce the risk of ocular invasion at the source has become a promising forward-looking prevention strategy [21,22]. In the present study, we constructed an animal model recapitulating authentic clinical pathogenic cascades to systematically evaluate the protective efficacy of PstS–YidR fusion mRNA vaccine against systemic infection, multi-organ injury, and endogenous endophthalmitis induced by K1 and K2 hvKp strains, providing experimental evidence to support further fundamental research and clinical translation of this vaccine.

We established an endogenous endophthalmitis mouse model via intraperitoneal bacterial challenge, which fully reconstructs the pathological chain of “abdominal infection → bacteremia → hematogenous dissemination → intraocular invasion” [23]. Compared with models relying on direct intravitreal injection or tail vein administration, this intraperitoneal infection model better mirrors the real pathogenesis of clinical endogenous eye infection [23]. Gradient dose screening confirmed 1.5 × 10^4^ CFU/mL as the optimal challenge concentration. Under this dosage, all mice survived throughout observation, and viable bacteria could be stably isolated from peritoneal lavage fluid, blood, liver and eyeball tissues, establishing a stable and reliable animal platform for vaccine efficacy assessment [24]. Consistent with clinical epidemiological evidence, our in vivo data demonstrated no significant disparities in pathogenicity, hematogenous dissemination capacity and intraocular invasiveness between K1 and K2 serotypes, which are widely recognized as the predominant hypervirulent serotypes worldwide [25,26,27].

Our phenotypic observations revealed that PstS–YidR fusion mRNA vaccination remarkably alleviated systemic infectious manifestations and stabilized body weight after bacterial challenge. Immunized mice only suffered transient mild discomfort and recovered within 3 days, a protective phenotype largely attributed to reduced bacterial colonization and systemic translocation post-vaccination. This cross-serotype protective activity against the tested K1 and K2 hypervirulent isolates is consistent with our conserved-antigen design strategy; however, the present in vivo study cannot fully separate antigen-specific effects from non-specific immune activation by the mRNA-LNP platform. PstS and YidR are two functionally conserved proteins with low sequence polymorphism across different K. pneumoniae serotypes, which is designed to overcome the serotype-limited protective defects of traditional capsular polysaccharide vaccines [28,29]. Additionally, the mRNA delivery platform is designed to facilitate persistent intracellular antigen expression, which may underlie robust in vivo antibacterial immunity [10]. Notably, all efficacy endpoints reported in this study are in vivo protective phenotypes; vaccine immunogenicity (antigen-specific antibody titers) was characterized in our previous publication [14] and was not re-evaluated here.

Multi-tissue bacterial culture, corroborated by per-animal 16S rRNA relative-abundance profiling, demonstrated that vaccination blocks hvKp translocation from the peritoneal cavity to blood, liver and ocular tissues and reduces bacterial loads across multiple organs, with intraocular bacterial relative abundance decreased by over 90% versus saline controls. These terminal-timepoint data demonstrate that vaccination reduces systemic bacterial burden and inhibits subsequent intraocular invasive events, which corresponds to the core pathogenic link of bacteremia-mediated ocular invasion in clinical endogenous endophthalmitis [2,30]. At day 38, vaccine intervention persistently restrained systemic bacterial colonization and lowered invasive bacterial loads in distant target organs such as the eye [31,32].

Intraocular inflammatory response and irreversible tissue destruction are the primary drivers of permanent vision loss in hvKp-related endophthalmitis [19]. Slit-lamp microscopic observation, semi-quantitative inflammatory scoring and histopathological staining at day 38 collectively validated that the vaccine attenuates intraocular inflammatory lesions and preserves the structural integrity of the cornea and retina in late-stage infection [19]. Vaccinated mice merely presented mild conjunctival congestion without vitreous purulent exudation, whereas unimmunized control animals exhibited typical suppurative pathological alterations including severe corneal edema, disrupted retinal architecture and massive vitreous inflammatory effusion [33]. These terminal observations suggest that the vaccine not only diminishes intraocular bacterial loads but is also associated with preservation of blood–ocular barrier integrity, plausibly via suppression of excessive inflammatory cascades at late infection stages. Hypervirulent *K. pneumoniae* disrupts endothelial cell junctions and elevates vascular permeability via multiple virulence factors; the blunted inflammatory response observed in vaccine groups alleviates such severe tissue destruction triggered by bacterial virulence mediators [34,35].

Because the challenge was non-lethal by design, the demonstrated efficacy reflects reduction in colonization, dissemination and ocular inflammatory burden rather than prevention of lethal sepsis—appropriate to the study’s aim of preventing endophthalmitis, in which colonization and intraocular invasion are the decisive events.

Uncontrolled inflammatory imbalance exacerbates multi-organ tissue injury after hvKp infection. Our CBA detection results confirmed that vaccination significantly downregulates tissue concentrations of IL-1β, IL-6 and TNF-α, limiting excessive release of pro-inflammatory mediators and subsequent organ damage [22]. The levels of these three cytokines in all sampled tissues of vaccinated mice only reached 18–19.5% of those measured in unvaccinated challenged animals, which greatly relieves cytokine-mediated inflammatory lesions across organs [36]. Hepatic histopathological results further supported the protective value of this vaccine: immunization decreased the incidence of liver abscess and mitigated massive hepatocellular necrosis, effectively blocking the classic clinical pathway of liver abscess complicated with secondary endogenous endophthalmitis [3].

The most prominent finding of this study is that the PstS–YidR fusion mRNA vaccine delivers equivalent cross-serotype protective effects against K1 and K2, the two most prevalent clinical hypervirulent serotypes. Traditional capsular polysaccharide vaccines only elicit serotype-specific protection and fail to meet the clinical demand for broad-spectrum defense against hvKp infections [37,38]. In contrast, the dual-conserved antigen design of our mRNA vaccine targets stable proteins shared among various serotypes and drug-resistant isolates, providing cross-serotype protective efficacy against the dominant clinical K1 and K2 hvKp strains and showing promising potential for prophylactic administration among high-risk clinical populations [10]. The antigen-specific basis of this protection, however, should be verified with empty-LNP or irrelevant-antigen platform controls that were not included in the present in vivo study.

In addition to prophylaxis, the mRNA–LNP platform might also be useful for intervention at early or post-exposure stages of hvKp infection, although its therapeutic use is limited by how fast protective immunity can be mounted. mRNA vaccines express antigen rapidly inside host cells and can be designed and produced much faster than conventional vaccines [9,10], which makes the platform well suited to outbreak-responsive deployment. For endogenous endophthalmitis, where retinal damage becomes irreversible within a narrow time window, and K. pneumoniae provokes a strong intraocular inflammatory cascade [18,34], the vaccine may be most helpful if given at the first signs of systemic infection or together with standard antibiotic and surgical treatment, potentially reducing hematogenous spread and intraocular inflammation. A practical constraint, however, is that de novo immune induction by mRNA still takes several days, whereas antibiotics kill bacteria immediately. The platform is therefore unlikely to replace antibiotics as first-line treatment for established or severe infection; if it has any therapeutic role, it would be in early or post-exposure settings, or in combination with antibiotics, rather than in fulminant disease. Direct testing of post-exposure or early-treatment dosing—preferably together with the antibiotic comparators mentioned in the Limitations—remains a task for future work.

Several limitations remain to be addressed in the current research. First, all protective efficacy assessments were performed on healthy laboratory mice, whereas most clinical high-risk patients carry underlying comorbidities including diabetes, immunosuppression and liver abscess, so vaccine performance in immunocompromised hosts needs further experimental verification. Accordingly, future efficacy studies should extend the evaluation to disease-relevant mouse models—such as streptozotocin-induced diabetic mice and immunocompromised strains (e.g., nude or SCID mice)—to verify protective performance in the high-risk populations that predominantly acquire hvKp infections in the clinic. Furthermore, the present study was designed to characterize the in vivo anti-infectious protective phenotypes of the PstS–YidR mRNA vaccine in the endogenous endophthalmitis model and deliberately did not re-measure vaccine immunogenicity. Humoral immunogenicity of this vaccine—namely high PstS-specific IgG titers and moderate YidR-specific IgG titers in mouse serum after prime-boost immunization—was established in our prior publication [14]. In the current work, we therefore did not perform supplementary detection of serum antigen-specific antibodies, mucosal IgA secretion, or T cell-mediated immune responses, and we could not quantify the independent contributions of humoral immunity, cellular immunity, and immune memory to overall protection. Importantly, the immunogenicity profiling in our previous study quantified binding antibody titers only and did not assess functional antibody activity; for Klebsiella pneumoniae defense, functional antibodies that mediate opsonization and complement-dependent killing are considered more predictive of protection than binding titers alone [12,29,31]. Direct functional assays such as opsonophagocytic killing or serum bactericidal assays were not conducted in either the prior or the present study. The absence of comprehensive immune and functional-antibody profiling limits our ability to infer the exact immune mechanisms and correlates of protection underlying the observed efficacy, and represents a key direction for dedicated follow-up work. To build a complete map of the vaccine-induced immune landscape, future immunogenicity profiling should quantify antigen-specific T-cell responses (e.g., IFN-γ ELISpot and intracellular cytokine staining for Th1/Th17 polarization), mucosal IgA at the relevant portals of entry (fecal/intestinal and respiratory-tract lavages), and memory B- and T-cell subsets, alongside the functional antibody assays noted above. Second, we only set a single terminal sampling time point (day 38 post-challenge) without serial tissue collection at early and middle infection stages to dynamically monitor fluctuations in bacterial loads and inflammatory mediators. Therefore, our dataset can only reflect protective phenotypes at late infection, unable to quantify the kinetics of bacterial clearance or dynamic tissue repair progress. Future studies will incorporate serial sampling at early and middle infection stages (e.g., days 1, 3, 7, 14, 21 and 28 post-challenge) to delineate the temporal trajectory of bacterial clearance and tissue repair. Third, this study exclusively explored prophylactic immunization effects, and we did not investigate the therapeutic potential of vaccine intervention at early infection timepoints. Fourth, an antibiotic treatment positive control group was not incorporated into our experimental design, making horizontal comparison between vaccine prophylaxis and antibiotic therapy impossible. These comparators should reflect clinically standard regimens for endogenous endophthalmitis and hvKp systemic infection—for example, intravitreal/intracameral vancomycin plus ceftazidime for ocular disease and systemic carbapenems or third-generation cephalosporins for disseminated infection. Subsequent research will establish independent or combined antibiotic intervention groups to supplement comparative experimental data. Fifth, the lack of an empty-LNP/irrelevant-antigen control means the observed protection cannot be fully separated from non-specific mRNA-LNP effects [14]. Sixth, cross-serotype protection was assessed with only one K1 (Kp. 8297) and one K2 (Kp. 9720) isolate, so broader coverage across diverse K. pneumoniae remains unproven. Seventh, bacterial burden in this study was quantified at the individual-animal level by 16S rRNA sequencing (per-mouse relative abundance, *n* = 5 per group), and culture plates were used as a qualitative readout of bacterial growth and the dominant colonizer rather than as a source of per-animal CFU counts. The 16S relative abundance measures relative, not absolute, tissue burden; absolute CFU quantification would require re-performance of culture enumeration or qPCR and is planned as follow-up work.

In summary, the PstS–YidR fusion mRNA vaccine effectively restricts systemic hvKp dissemination, reduces intraocular invasion risk, alleviates multi-organ pathological injury and restores inflammatory homeostasis. It provides equivalent cross-serotype protection against K1 and K2 strains with favorable in vivo safety profiles. Distinct from traditional serotype-restricted vaccines, this candidate mRNA vaccine exhibits preventive value against drug-resistant hypervirulent *K. pneumoniae* and its ocular complications, offering a novel prophylactic strategy for clinical high-risk populations. This work possesses important scientific significance and clinical translational prospects for lowering the incidence of endogenous endophthalmitis, reducing permanent visual impairment, and alleviating global antimicrobial resistance threats.

## 5. Conclusions

The results of this study demonstrate that the PstS-YidR fusion mRNA vaccine delivers prominent prophylactic protective effects against systemic disseminated infection and endogenous endophthalmitis triggered by hypervirulent K1 and K2 serotypes of *K. pneumoniae*. The vaccine suppresses bacterial colonization and systemic spread across the peritoneal cavity, blood, liver, lung and ocular tissues, lowers tissue bacterial loads, and attenuates systemic inflammatory activation and intraocular inflammatory lesions. Equivalent protective outcomes against the two dominant hypervirulent serotypes are achieved across the two tested hypervirulent serotypes, with favorable in vivo safety profiles. Collectively, the PstS-YidR fusion mRNA vaccine represents a cross-serotype protective candidate against the dominant clinical K1 and K2 hvKp isolates, providing a novel prophylactic strategy for severe infectious diseases and endogenous endophthalmitis induced by hypervirulent *K. pneumoniae*, pending rigorous validation with empty-LNP/irrelevant-antigen platform controls.

## Figures and Tables

**Table 1:** Experimental groups.

Group No.	Group Name	Day 0 i.m. Injection	Day 21 i.m. Injection	Day 35 i.p. Challenge
A	Blank control group	–	–	–
B	Vaccine-Kp.1 group	Vaccine	Vaccine	Kp.8297 suspension
C	Vaccine-Kp.2 group			Kp.9720 suspension
D	NaCl-Kp.1 group	0.9% NaCl	0.9% NaCl	Kp.8297 suspension
E	NaCl-Kp.2 group			Kp.9720 suspension

Note: i.m., intramuscular; i.p., intraperitoneal; “–”, no treatment.

**Table 2:** Survival of mice 4 days after intraperitoneal challenge with graded doses of K1 and K2 hypervirulent K. pneumoniae.

Group	Injection Concentration (CFU/mL)	Time Range of Death	Number of Survivors (*n*)	Survival Rate (%)
Control group	–	–	10	100
hvKp.1 group	1.5 × 10^2^	–	10	100
	1.5 × 10^4^	–	10	100
	1.5 × 10^6^	Day 1–2	6	60
	1.5 × 10^8^	Day 2–3	2	20
hvKp.2 group	1.5 × 10^2^	–	10	100
	1.5 × 10^4^	–	10	100
	1.5 × 10^6^	Day 1–2	6	60
	1.5 × 10^8^	Day 2–3	2	20

Note: “–”, no mortality observed within the 4-day observation period; *n* = 10 mice per subgroup.

**Table 3:** Semi-quantitative scoring of ocular inflammation in mice on D38 (mean ± SD, *n* = 5).

Item	Control	Vaccine-Kp.1	Vaccine-Kp.2	NaCl-Kp.1	NaCl-Kp.2	F	*p*
Congestion	0.00 ± 0.00	1.20 ± 0.42	1.30 ± 0.48	2.30 ± 0.48	2.20 ± 0.42	92.68	<0.001
Cornea	0.00 ± 0.00	0.00 ± 0.00	0.00 ± 0.00	2.70 ± 0.48	2.60 ± 0.55	145.31	<0.001
Iris	0.00 ± 0.00	0.50 ± 0.53	0.60 ± 0.55	2.80 ± 0.42	2.70 ± 0.48	101.57	<0.001
Vitreous	0.00 ± 0.00	0.00 ± 0.00	0.00 ± 0.00	2.60 ± 0.55	2.50 ± 0.53	138.64	<0.001
Total	0.00 ± 0.00	1.70 ± 0.76	1.90 ± 0.83	10.40 ± 1.35	10.00 ± 1.27	235.79	<0.001

Note: Scoring criteria: 0 = none/normal; 1 = mild; 2 = moderate; 3 = severe. Congestion (conjunctival congestion), Cornea (corneal opacity), Iris (iris inflammation), Vitreous (vitreous opacity). Total score: 0–12.

## Data Availability

The original contributions presented in this study are included in the article. Further inquiries can be directed to the corresponding authors.
